# Fracture propagation and pore pressure evolution characteristics induced by hydraulic and pneumatic fracturing of coal

**DOI:** 10.1038/s41598-024-60873-2

**Published:** 2024-05-01

**Authors:** Cao Zhengzheng, Yang Xiangqian, Li Zhenhua, Huang Cunhan, Du Feng, Wang Wenqiang, Ni Xianjie, Liu Shuai, Li Zhen

**Affiliations:** 1 International Joint Research Laboratory of Henan Province for Underground Space Development and Disaster Prevention, School of Civil Engineering, Henan Polytechnic University, Jiaozuo, 454000 Henan China; 2 Henan Mine Water Disaster Prevention and Control and Water Resources Utilization Engineering Technology Research Center, Henan Polytechnic University, Jiaozuo, 454000 Henan China; 3 Collaborative Innovation Center of Coal Work Safety and Clean High Efficiency Utilization, Jiaozuo, 454000 Henan China; 4 State Key Laboratory of Mining Response and Disaster Prevention and Control in Deep Coal Mines, Anhui University of Science and Technology, Huainan, 232001 Anhui China; 5 China Coal Xinji Energy Limited Liability Company, Huainan, 232001 Anhui China; 6 College of Safety and Emergency Management Engineering, Taiyuan University of Technology, Taiyuan, 030024 Shanxi China

**Keywords:** Rock mechanics, Hydraulic fracturing, Pneumatic fracturing, Pore pressure, Acoustic emission, Coal, Civil engineering

## Abstract

A two-dimensional unsteady seepage model for coal using a finite element program is developed, and the temporal variations of key factors such as water pressure and hydraulic gradient are analyzed in this paper. Additionally, the triaxial rock mechanical experiment and utilized pneumatic fracturing equipment on raw coal samples to investigate both hydraulic and pneumatic fracturing processes are conducted. Through these experiments, the relationship between pressure and crack formation and expansion are examined. The analysis reveals that the pore pressure gradient at the coal inlet reaches its peak during rapid surges in water pressure but diminishes over time. Conversely, the pore pressure gradient at the outlet side exhibits a gradual increase. Hydraulic fracturing is most likely to occur at the water inlet during sudden increases in water pressure. Besides, as the permeability of coal decreases, the duration for seepage stabilization prolongs due to the intensified pore pressure gradient resulting from sudden increases in water pressure. Moreover, an extended period of high hydraulic gradient further increases the risk of hydraulic fracturing. The experimental findings indicate that coal samples initially experience tensile failure influenced by water and air pressure. Subsequently, mode I cracks form under pressure, propagating along the fracture surface and becoming visible. The main types of failure observed in hydraulic and pneumatic fracturing are diametrical tensile failure, and the development of fractures can be categorized into three distinct stages, which contains the initial stage characterized by slight volume changes while water pressure increases, the expansion stage when pressure reaches the failure strength, and the crack closure stage marked by little or even decreasing volume changes during pressure unloading. The acoustic emission signal accurately corresponds to these three stages.

## Introduction

Hydraulic fracturing is a kind of performance of seepage-stress coupling, which reflects the mechanical response and structural changes of rock and soil under the action of seepage water^1^. This phenomenon refers to the occurrence and propagation of cracks in rocks and soils under the influence of significant differences in water pressure^2^. Since its successful implementation in the United States in 1947, hydraulic fracturing has become widely adopted in the modern petroleum industry as the primary technique for enhancing production in oil fields and mines^3^. Nowadays, hydraulic fracturing method is widely used in geotechnical and hydraulic engineering. In the realm of geotechnical engineering, hydraulic fracturing technology has attained a commendable level of maturity, particularly in its application for in-situ stress measurements^4,5^. However, hydraulic fracturing not only brings benefits, but also brings disasters, such as mine water inrush, large amount of water inrush in tunnel construction and operation, water storage cracking of reservoir dam, etc^6,7^. Therefore, it holds an significant importance for both engineering and academic communities to investigate the mechanism underlying water-induced fracturing.

Hydraulic fracturing technology has been used in modern petroleum industry, geothermal resources development and other fields, showing a wide range of industrial application value^8,9^. Pneumatic fracturing is similar to hydraulic fracturing principle, which compresses the gas to form high-pressure gas and cracks the coal seam, so as to realize coal seam permeability enhancement and gas drainage under the condition of no water resources. Understanding the mechanism behind hydraulic and pneumatic fracturing in gas drainage holds immense significance. Many scholars have studied hydraulic and pneumatic fracturing. Xu et al.^10^ carried out deformation test of sandstone under cyclic water pressure by MTS815 rock mechanics multifunctional testing machine. Deng et al.^11–13^ studied the control parameters of the propagation behavior of water pressure crack for large coal samples, and studied the mechanical mechanism of water pressure crack destroying coal structure, which provides a scientific basis for solving the control of the propagation behavior of water pressure crack in coal medium materials. Ding and Sun^14^ obtained linear elastic stress solutions for thick-walled cylinders under three distinct conditions: steady seepage, isotropic consolidation, and non-isotropic consolidation. They believed that the hydraulic fracturing was caused by the tensile failure at the inner hole wall first, and then extended outwards until the crack penetrated. According to the stress state of the hole wall in the fractured section, Yang et al.^15^ conducted an analysis on the fracture mechanism of hydraulic fracturing, examining both the tensile fracture criterion and Mohr Coulomb criterion. They established that there are two distinct fracture modes: tensile fracture and shear fracture. Zhan and Cen^16^ proposed an action mechanism, winch takes into account the seepage force based on hydraulic splitting tests conducted on thick-walled cylinder specimens. They derived an analytical solution for the stress distribution in thick-walled cylinders subjected to stable seepage conditions. They conducted a verification process which established that hydraulic splitting corresponds to tensile failure, specifically when the stress exerted on the inner ring reaches the ultimate tensile strength of the specimen. Yang et al.^17^ studied it from the aspect of fracture mechanics. Wang Yuan et al.^18^ developed a one-dimensional unsteady seepage model and derived its analytical solution. They further compared the temporal changes in hydraulic pressure and hydraulic gradient, crucial factors in hydraulic fracturing, with the results obtained from finite element simulations.

Hydraulic fracturing in coal mining exemplifies the influence of seepage water on the structural integrity of coal formations. In the traditional study of hydraulic fracturing, the effect of seepage was not considered. Later, the importance of seepage was realized, For example, Ding et al.^14^ carried out hydraulic fracturing test on hollow cylinder specimen under the condition of stable seepage. By utilizing Darcy's law and the equilibrium equation, the researchers derived the expressions for the radial stress and circumferential stress experienced by the specimen under the influence of seepage force. Furthermore, they compared the theoretical fracturing pressure with the corresponding experimental value. It is proved that the study of hydraulic fracturing mechanism should be considered from the perspective of permeability. Using thick wall cylinder test, Zhan et al.^16^ pointed out that the analysis method based on lame formula was wrong, and proposed to study hydraulic fracturing from the mechanism of seepage force, but only the state of stable seepage was considered. Wang et al.^18,19^ developed a one-dimensional unsteady seepage model, derived an analytical solution for this model, and subsequently compared the temporal variation patterns of both water pressure and hydraulic gradient-crucial factors in hydraulic fracturing-with the results obtained from finite element simulations. However, only one-dimensional unsteady seepage state was considered, while in practical engineering, two-dimensional and three-dimensional problems were mostly considered. In terms of stress mechanism, hydraulic fracturing causes cracks in rock mass, which is essentially related to seepage force caused by seepage. The permeability is the reflection of the pressure gradient of pore water^20–23^. Therefore, the occurrence of hydraulic fracturing is determined not only by water pressure but also by the proximity of the hydraulic gradient to the potential splitting surface.

## Analysis of coal fracture formation process

**Figure 1:**
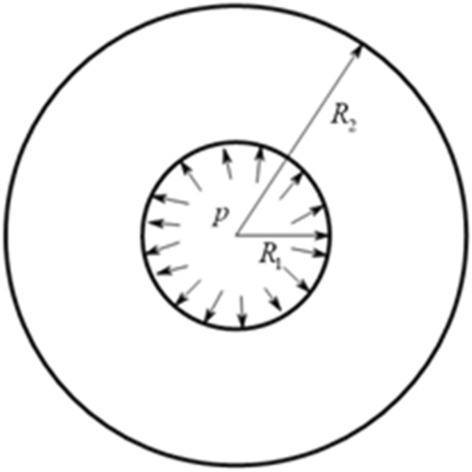
Cylinder model.

According to the shape of the experimental coal sample, the sample is simplified as a cylinder, as shown in Fig. 1.

Therefore, the boundary conditions require:

1 $$ \begin{gathered} (\tau_{\rho \varphi } )_{{\rho = R_{1} }} = 0 \hfill \\ (\tau_{\rho \varphi } )_{{\rho = R_{2} }} = 0 \hfill \\ (\sigma_{\rho } )_{{\rho = R_{1} }} = - p \hfill \\ \end{gathered} $$

where the meaning in the Eq. (1) is shown in Fig. 1. The expression of stress in surrounding rock of borehole wall can be written as:

2 $$ \sigma_{\rho } = - \frac{{\frac{{R_{2}^{2} }}{{r^{2} }} - 1}}{{\frac{{R_{2}^{2} }}{{R_{1}^{2} }} - 1}}p,\quad \sigma_{\varphi } = \frac{{\frac{{R_{2}^{2} }}{{r^{2} }} + 1}}{{\frac{{R_{2}^{2} }}{{R_{1}^{2} }} - 1}}p $$

In the failure analysis of coal sample, the coal sample is simplified as an infinite plane circular hole subjected to uniform force. Coulomb criterion is used to judge the failure of coal sample, and Coulomb criterion is used to calculate the dip angle of shear fracture section. In this paper, two failure criteria are used to verify the above conclusions.

1. Failure criterion of maximum tensile stress.
  3 $$ F = \sigma - T_{0} (k) = 0 $$
  where $T_{0}$ is the tensile strength of the material under tension.
2. Coulomb criterion. The main point of the code is that the failure of coal is shear failure.

**Figure 2:**
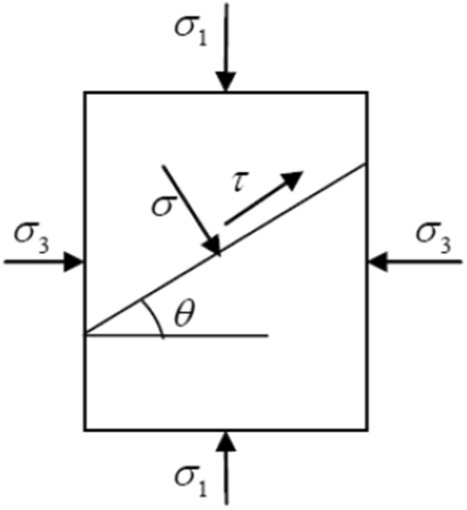
Force analysis of micro-element slope.

Take the cross section of the cylinder model and take a micro element, as shown in Fig. 2. The pressure is positive.

It can be seen that the stress of micro-elements is

4 $$ \sigma_{1} = \frac{{\frac{{R_{2}^{2} }}{{r^{2} }} - 1}}{{\frac{{R_{2}^{2} }}{{R_{1}^{2} }} - 1}}p,\;\sigma_{3} = - \frac{{\frac{{R_{2}^{2} }}{{r^{2} }} + 1}}{{\frac{{R_{2}^{2} }}{{R_{1}^{2} }} - 1}}p $$

where the meaning in the Eq. (4) is shown in Fig. 2. The shear strength criterion in plane is as follows:

5 $$ \{ \left| \tau \right| - f\sigma \}_{\max } = \frac{1}{2}(\sigma_{1} - \sigma_{3} )\sqrt {f^{2} + 1} - \frac{1}{2}f(\sigma_{1} + \sigma_{3} ) $$

where $f = \tan \varphi$. According to the equation, if the equation is less than $c$, the failure can not occur; If it is equal to (or greater than) $c$, then failure occurs. The complete strength curve of Coulomb criterion in coordinate system is:

6 $$ \left\{ {\begin{array}{*{20}c} {\sigma_{1} [\sqrt {f^{2} + 1} - f] - \sigma_{3} [\sqrt {f^{2} + 1} + f] = 2c} & {(\sigma_{1} > \frac{1}{2}\sigma_{c} )} \\ {\sigma_{3} = - \sigma_{1} } & {(\sigma_{1} \le \frac{1}{2}\sigma_{c} )} \\ \end{array} } \right. $$

7 $$ \sigma_{c} = 2c[\sqrt {f^{2} + 1} + f] $$

According to the calculation of coal sample parameters, the coal sample does not reach the shear failure of Coulomb criterion, but directly reaches the maximum tensile strength failure. However, in the experiment, the failure of the coal sample becomes diametrical tensile failure, which is two vertical lines with small dip angle. Therefore, after the cylindrical coal sample is subjected to water pressure and air pressure, the tensile stress of the inner wall boundary is the largest. Due to the heterogeneity of the coal, the tensile crack is firstly carried out at the weak part of the structure.

**Figure 3:**
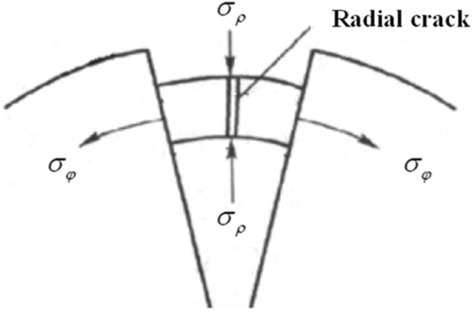
Radial fracture propagation process.

Coal is not isotropic material, it is an an-isotropic complex combination, in the process of formation, there are many defects, these cracks change the mechanical properties of coal. As shown in Fig. 3, the main body of the device is destroyed and radial cracks are formed.

## Analysis of coal fracture propagation process

When the coal sample reaches the tensile stress strength, it is destroyed and cracks appear. Next, the crack propagation is studied. The hydraulic crack in the hole wall is tensile crack. According to the theory of fracture mechanics, the mode I crack fracture model can be effectively employed to analyze the propagation characteristics of coal following the occurrence of cracks.

**Figure 4:**
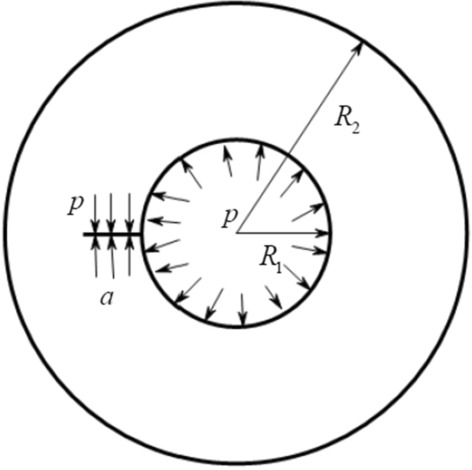
Circular hole model with crack length a.

As illustrated in Fig. 4, the model is simplistically represented as a crack of length a within the internal pressure cylinder. The cylinder exhibits an inner radius denoted as *R*_1_, an outer radius denoted as *R*_2_, and the depth of the crack on the inner wall is determined as *a*. Additionally, the pressure within the cylinder is designated as *p*.

Checking the "stress intensity factor manual", the stress intensity factor at the crack front is

8 $$ K_{{\text{I}}} = F\;\sigma \sqrt {\pi a} = \frac{{2FpR_{2}^{2} \sqrt {\pi a} }}{{R_{2}^{2} - R_{1}^{2} }} $$

9 $$ K_{{{\text{II}}}} = K_{{{\text{III}}}} = 0 $$

The value of $F$ can be obtained by looking up the table. When the crack is very shallow ($a \to 0$), $\mathop {\lim }\limits_{a \to 0} K_{{\text{I}}} = 1.12\;\sigma \sqrt {\pi a}$.

The criterion of initial state of fracture propagation is as follows:

10 $$ K_{{\text{I}}} = F\;\sigma \sqrt {\pi a} = \frac{{2FpR_{2}^{2} \sqrt {\pi a} }}{{R_{2}^{2} - R_{1}^{2} }} \ge K_{{{\text{I}}d}} $$

The formula incorporates the dynamic fracture toughness of the initial coal, denoted as $K_{{{\text{I}}d}}$. The magnitude of $K_{{{\text{I}}d}}$ directly correlates with the coal material's resistance to fracture—a higher value indicates a greater ability to withstand fracture. This intrinsic characteristic can be quantified through experimental measurement.

The results are as follows:

11 $$ p \ge \frac{{K_{{{\text{I}}d}} (R_{2}^{2} - R_{1}^{2} )}}{{2FR_{2}^{2} \sqrt {\pi a} }} $$

It is not difficult to find from the above formula that assuming that the shape of coal remains unchanged, the driving pressure required is smaller and smaller with the increasing crack length of crack *a*, and the crack grows exponentially with the continuous influx of gas. This is also consistent with the experimental phenomenon. After the crack appears, the crack propagation takes place in a short time, resulting in diametral tension crack. Then, due to the unloading of water pressure and air pressure, the crack growth stops.

## Two dimensional hydraulic fracturing unsteady seepage model and analytical solution

### Two dimensional unsteady seepage model of coal

**Figure 5:**
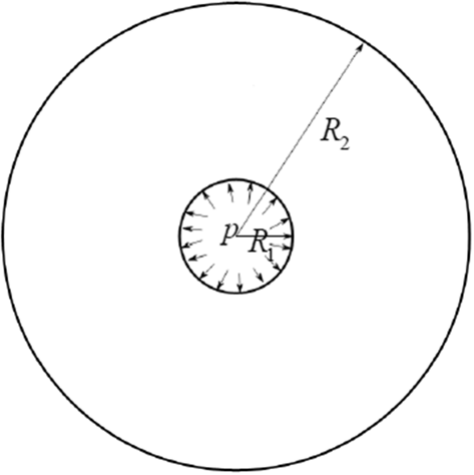
Two dimensional coal sample model.

Take a thin uniform disk with radius 1, let it be isotropic, the initial water pressure is *p*_0_ = 0, and apply water pressure *p* at the center. The changes of pore pressure and pore pressure gradient at any place in coal with time at *t* time are investigated to analyze the hydraulic fracturing process of standard coal samples under laboratory conditions. In Fig. 5, it is evident that the initial pore pressure state within the model remains uniform across all points.The initial condition is *p*(*r*, 0) = 0. The water level at the center rises rapidly, and the water pressure changes from *p*_0_ to *p*_1_ in a moment, and the water pressure at the exit is still kept at *p*_0_ = 0. The water flow movement in this model can be expressed as follows:

12 $$ \nabla^{2} p = \frac{{\phi \mu C_{t} }}{K}\frac{\partial p}{{\partial t}} $$

where the variable $K$ represents permeability, $\mu$ denotes viscosity, and $\phi$ signifies the porosity of the coal sample, $C_{t}$ is comprehensive compressibility.

### Analytical solution of seepage field of 2D hydraulic fracturing model

As the problem necessitates a precise solution for a circular context, the adoption of a polar coordinate system becomes pertinent. Consequently, the coordinate origin is established at the center of the disk. In order to solve the problem conveniently, the initial $p(r,0) = f(r)$ is assumed to be independent of the variable $\theta$, and the pressure is centrosymmetric when it changes with time, so the problem of definite solution becomes

13 $$ \begin{gathered} \frac{\partial p}{{\partial t}} = a^{2} (\frac{{\partial^{2} p}}{{\partial r^{2} }} + \frac{1}{r}\frac{{\partial^{2} p}}{\partial r})\quad (0 < r < 1,t > 0) \hfill \\ p(1,t) = 0\quad p(0,t) = p\quad (t > 0) \hfill \\ p(r,0) = f(r)\quad \quad \quad \quad \;\;(0 \le r \le 1) \hfill \\ \end{gathered} $$

Consider the aforementioned equation, which can be resolved through the utilization of separated variables, yielding a formal solution

14 $$ p(r,t) = R(r)T(t) $$

Obtain that

15 $$ RT^{\prime} = a^{2} (R^{\prime\prime}T + \frac{1}{r}R^{\prime}T) $$

From this a conclusion can be drawn

16 $$ T^{\prime}{ + }\lambda a^{2} T = 0 $$

17 $$ r^{2} R^{\prime\prime}{ + }rR^{\prime} + \lambda r^{2} R = 0 $$

The solution of Eq. (16) is

18 $$ T(t) = {\text{e}}^{{ - a^{2} \lambda t}} $$

Since $p$ is a finite value when $t \to \infty$, $\lambda$ is a positive value. Take $\lambda { = }\mu^{2}$, then

19 $$ T(t) = {\text{e}}^{{ - a^{2} \mu^{2} t}} $$

Equation (17) is the zero order Bessel equation, and its general solution is

20 $$ R(r) = AJ_{0} (\mu r) + BY_{0} (\mu r) $$

Since the Bessel function a of the second kind $Y_{0}$ is not bounded at $r = 0$, $B{ = }0$ must be taken, so the above formula becomes

21 $$ R(r) = AJ_{0} (\mu r) $$

From the boundary conditions

22 $$ J_{0} (\mu ){ = }0 $$

It can be seen that $\mu$ is the positive zero solution of zero order Bessel function, that is, $\mu { = }\mu_{0m} (m = 1,2,3, \cdots )$. In conclusion, the results can be obtained

23 $$ R_{m} (r) = A_{m} J_{0} (\mu_{0m} r) $$

24 $$ T_{m} (t) = {\text{e}}^{ - a^{2} {\mu_{0m }^2} t} $$

Thus the eigen solution is

25 $$ p_{m} (r,t) = A_{m} J_{0} (\mu_{0m} r){\text{e}}^{ - a^{2} \mu_{0m }^2 t} $$

By using the superposition principle, the general solution of the definite solution problem is obtained

26 $$ p(r,t) = \sum\limits_{m = 1}^{\infty } {A_{m} J_{0} (\mu_{0m} r){\text{e}}^{ - a^{2} \mu_{0m }^2 t} } $$

Using the initial conditions to determine the coefficient $A_{m}$, there are

27 $$ f(r) = \sum\limits_{m = 1}^{\infty } {A_{m} J_{0} (\mu_{0m} r)} $$

It can be obtained from the expansion of function $f(r)$

28 $$ A_{m} { = }\frac{2}{{J_{1}^{2} (\mu_{0m} )}}\int_{0}^{1} {xJ_{0} (\mu_{0m} x)f(x)} {\text{d}}x $$

The pore pressure distribution is obtained

29 $$ p(r,t) = \sum\limits_{m = 1}^{\infty } {A_{m} J_{0} (\mu_{0m} r){\text{e}}^{ - a^{2} \mu_{0m }^2 t} } $$

This is the solution of the problem, where $A_{m} { = }\frac{2}{{J_{1}^{2} (\mu_{0m} )}}\int_{0}^{1} {xJ_{0} (\mu_{0m} x)f(x)} {\text{d}}x$, $\mu { = }\mu_{0m} (m = 1,2,3, \cdots )$ is the $m$ positive zero of $J_{0} (x)$.

## Analysis of the influential mechanisms pertaining to the unsteady seepage in hydraulic fracturing

The preliminary analytical solution of the two-dimensional unsteady seepage model of coal is obtained above, which can not be expressed by finite elementary functions and is difficult to be solved further. Therefore, the commercial finite element software COMSOL is employed to simulate the hydraulic fracturing process of coal samples under laboratory conditions, as shown in the Fig. 5, $R_{1} = {\text{5\,  mm}}, R_{2} = 2{\text{5\,  mm}}, p = 3\, {\text{ MPa}}$, Outer boundary $p_{0} = 0$. Take $K{ = 1} \times 10^{{{ - }17}}$ m^2^, $\mu { = }1 \times 10^{{{ - }17}}\, {\text{m}}^{2}$, $\phi { = }0.03$.

### Variation of pore pressure and pore pressure gradient at different positions with time

**Figure 6:**
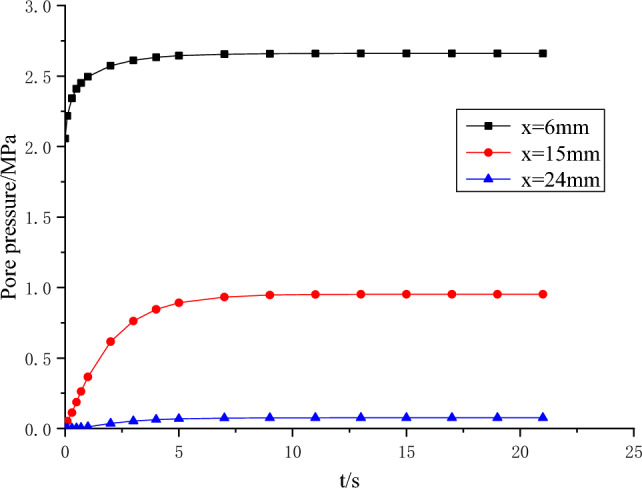
Relationship curve between pore pressure and time.

**Figure 7:**
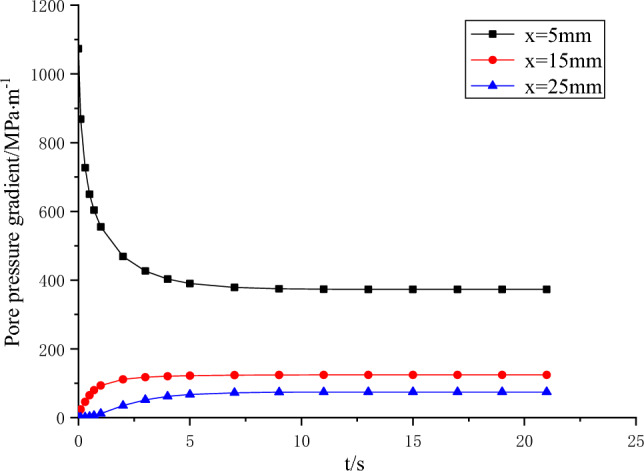
Relationship curve between pore pressure gradient and time.

The curves of calculated pore pressure and pore pressure gradient versus time are shown in Figs. 6 and 7, respectively.

Figure 6 illustrates the variation in pore pressure at distances of 6 mm, 15 mm, and 24 mm. Notably, it is observed that under conditions of unsteady seepage, smaller coal sizes transition to a stable state relatively quickly. Furthermore, it is worth noting that the pressure increment is more prominent in the vicinity of the water inlet. In Fig. 7, it is evident that an initial significant void pressure gradient emerges in proximity to the water inlet, which subsequently stabilizes over time. Near the outer boundary, the void pressure gradually increases from zero to steady state in a short time. This phenomenon can be attributed to the rapid increase in water pressure at the inlet, which restricts its outward penetration and consequently establishes a steep pressure gradient. As time progresses, the water near the inlet gradually permeates towards the outlet, resulting in a decrease in the pressure gradient, while the pressure itself continues to increase.

Therefore, it can be considered that the possibility of hydraulic fracturing is the largest when the initial water pressure increases suddenly, and the possibility of hydraulic fracturing becomes smaller when the seepage tends to be stable and the water pressure gradient decreases gradually. In practice, the coal sample is fractured at the moment when the water pressure increases, not when the seepage reaches stability.

### Relationship between pore pressure and pore pressure gradient at different time

**Figure 8:**
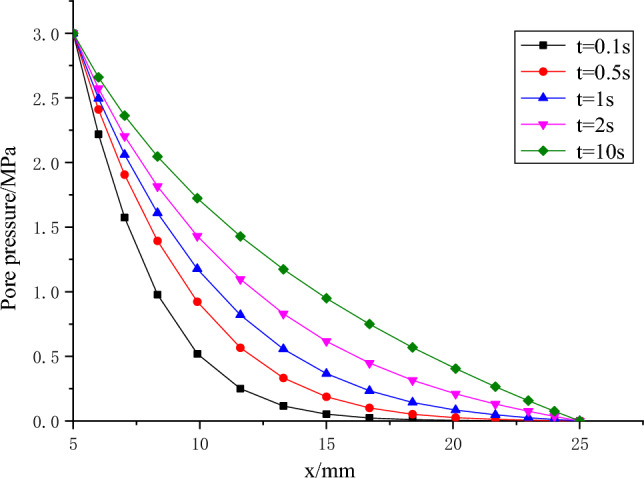
Variation of pore pressure on the whole specimen at different times.

**Figure 9:**
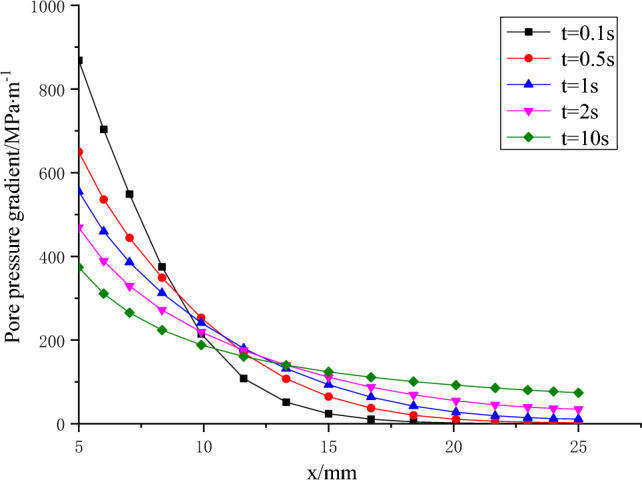
Variation of pore pressure gradient on the whole specimen at different times.

Figure 8 displays the temporal variation of pore pressure across the entire specimen. Observations from the figure indicate a progressive increase in pore pressure at each point as time advances, ultimately reaching a state of equilibrium. Figure 9 illustrates the temporal evolution of the pore pressure gradient across the entire specimen. As time progresses, the pore pressure gradient on the inlet side steadily decreases while the gradient on the outlet side gradually increases. This observation indicates that the period of greatest hydraulic fracturing risk coincides with the sudden surge in water pressure at the water inlet.

### Relationship between pore pressure and pore pressure gradient of coal samples with different permeability

**Figure 10:**
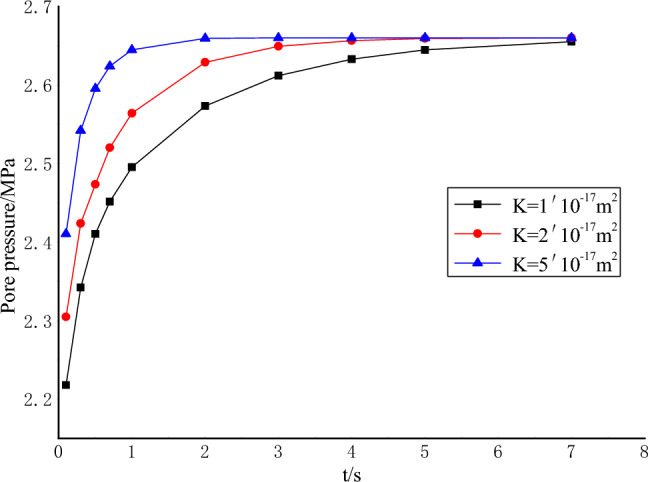
Relationship between pore pressure and time at *x* = 6 mm.

**Figure 11:**
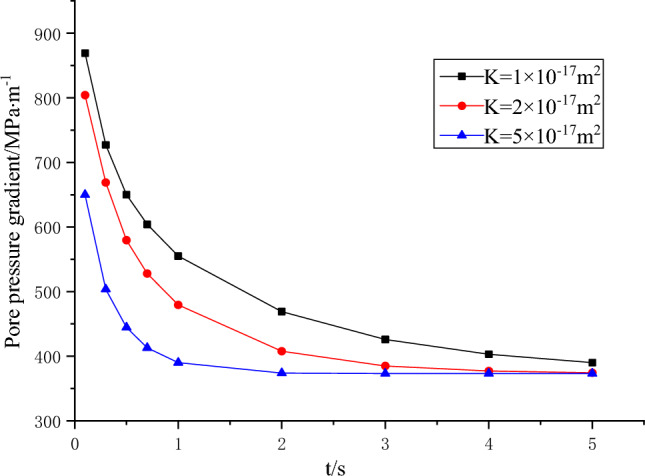
Variation of pore pressure gradient with time at *x* = 5mm.

The pressure at the water intake is *p* = 3MPa, and the comparative analysis is carried out according to different coal sample permeability $K{ = 1} \times 10^{{{ - }17}}$ m^2^, ${2} \times 10^{{{ - }17}}$ m^2^ and ${5} \times 10^{{{ - }17}}$ m^2^. Figure 10 depicts the temporal relationship between pore pressure and time at a specific location, *x* = 6 mm. Figure 11 illustrates the temporal correlation between pore pressure gradient and time at a specific position, *x* = 5 mm.

As depicted in Fig. 10, it is evident that a lower permeability corresponds to a slower propagation of water pressure from the water inlet to the water outlet during sudden increases in water pressure. Additionally, lower permeability results in lower pore pressure in close proximity to the water inlet. Over time, there is a gradual and ultimately stable increase in pore pressure. It is worth noting that the stabilization of pore pressure is delayed in materials with lower permeability. Figure 11 reveals a clear relationship between permeability and the pore pressure gradient at the water inlet. Specifically, a smaller permeability is associated with a higher pore pressure gradient. As time progresses, the pore pressure gradient exhibits a gradual decline and reaches a state of stability. Notably, materials with lower permeability require a longer duration to achieve this stable state. Therefore, it can be inferred that the spatiotemporal dynamics of pore pressure and its gradient are directly associated with the permeability properties. A lower permeability results in a diminished rate of outward pore pressure propagation at the coal sample inlet. Simultaneously, it leads to an increased pore pressure gradient and an extended duration until seepage in the coal sample attains stability. Consequently, this implies a prolonged period of high water pressure gradient.

## Test analysis

All the samples are processed in the natural dry and wet state, and the characteristics of raw coal should be kept as far as possible. The specimens are international standard cylindrical specimens ( *Φ* = 50mm, *H* = 100mm, and the end face parallelism is controlled within ± 0.02 mm). A cylindrical hole, measuring 30mm in depth and 5mm in diameter, is precisely drilled at the center of both end faces of the coal sample. So as to connect the external high-pressure gas (liquid) pipe during the experiment. During the experiment, glass sealant and heat shrinkable film are used to prevent coal sample from being immersed in oil.

This test procedure is finished with reference to the references^1–3,21^. Rock mechanics test system is used in the experiment. The tester can automatically perform various tests, such as uniaxial compression, triaxial compression, pore permeability and direct shear. After self-improvement, the testing machine is connected with the air compressor and supplemented with pressure monitor, forming a complete set of high-pressure gas and high-pressure water servo test system. High pressure gas (high pressure water) fracture and fatigue test can be completed more accurately. The acoustic emission signal of the coal sample is tested using an electrical data acquisition system during the experiment.

The tester is equipped with a load control method, which is added to 8kN at the speed of 40N/s and remains unchanged. Control the pressure of gas (liquid) until the coal sample is destroyed. Because of the fracture of brittle rock, the occurrence and propagation of cracks can be monitored by acoustic emission, and the development of cracks on the sample surface can be observed.

### Deformation characteristics of fractured coal

**Figure 12:**
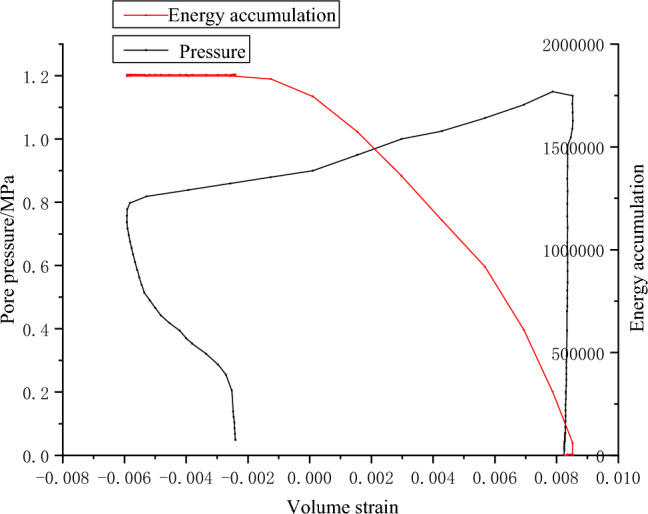
Pressure–volume strain relationship and AE energy accumulation curve of coal sample G1.

**Figure 13:**
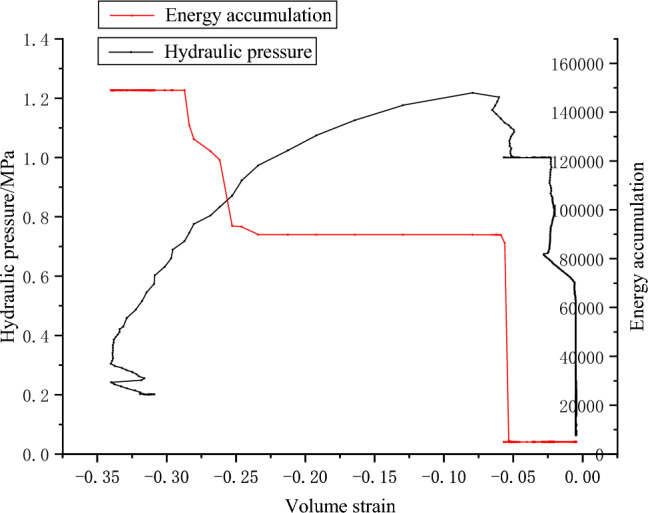
Water pressure–volume strain relationship and AE energy accumulation curve of coal sample W1.

**Figure 14:**
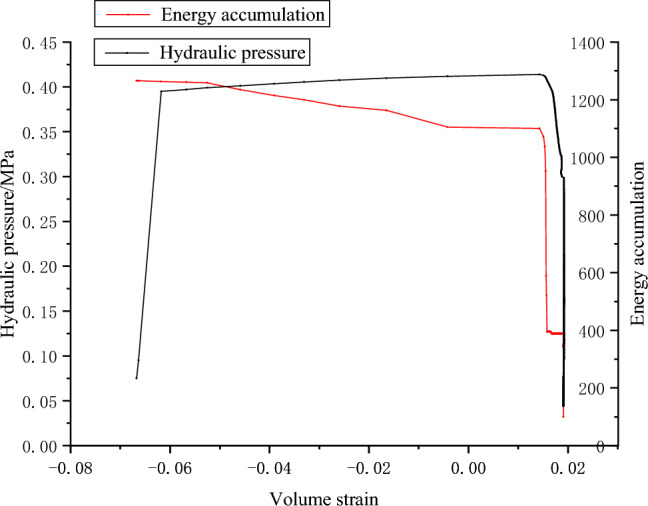
Water pressure–volume strain relationship and AE energy accumulation curve of coal sample W3.

The acoustic emission parameters of coal samples are obtained by fracturing test of raw coal water pressure, as shown in Figs. 12, 13 and 14.

In the process of hydraulic fracturing, complex permeability changes occur in coal under complex stress. The failure mode of the coal sample is evident in sample W1, where significant volume expansion occurs both during and after the fracturing process. The magnitude of surface volumetric strain exhibits a larger variation as the slope of the curve decreases. There are three main stages of coal cracking. Specifically, the first stage is that the pressure increases gradually, the volume strain changes little and forms a vertical line. The second stage is when the pressure reaches the maximum fracture pressure and limit pressure of coal system. In the hydraulic fracturing test of sandstone, because of the brittleness and good permeability of sandstone, there is obvious cracking sound at the moment of failure, while in the fracturing test of coal sample, the coal sample itself has no obvious observation characteristics, and the failure of coal sample can be monitored by acoustic emission and hydraulic unloading. At this stage, due to the coal sample cracks, water flows in the fracturing channel, and the water pressure begins to unload. The volume strain of coal sample increases rapidly. In a few seconds, the water pressure has not been fully unloaded, and the slope of the curve is small, which indicates that the volume expansion is large in a short time when the water pressure changes. Then, in the third stage, the water pressure gradually unloads, and the volume strain increased slowly. After the water pressure unloading, the pressure of G1 and W1 decreases, the fracture closes, and the volume strain decreases. Due to the unloading of osmotic pressure, part of the fracture is closed elastically. The acoustic emission signal effectively characterizes the three stages. During the first and third stages, the energy counting rate remains relatively low, whereas in the second stage, there is a significant increase in energy accumulation.

**Figure 15:**
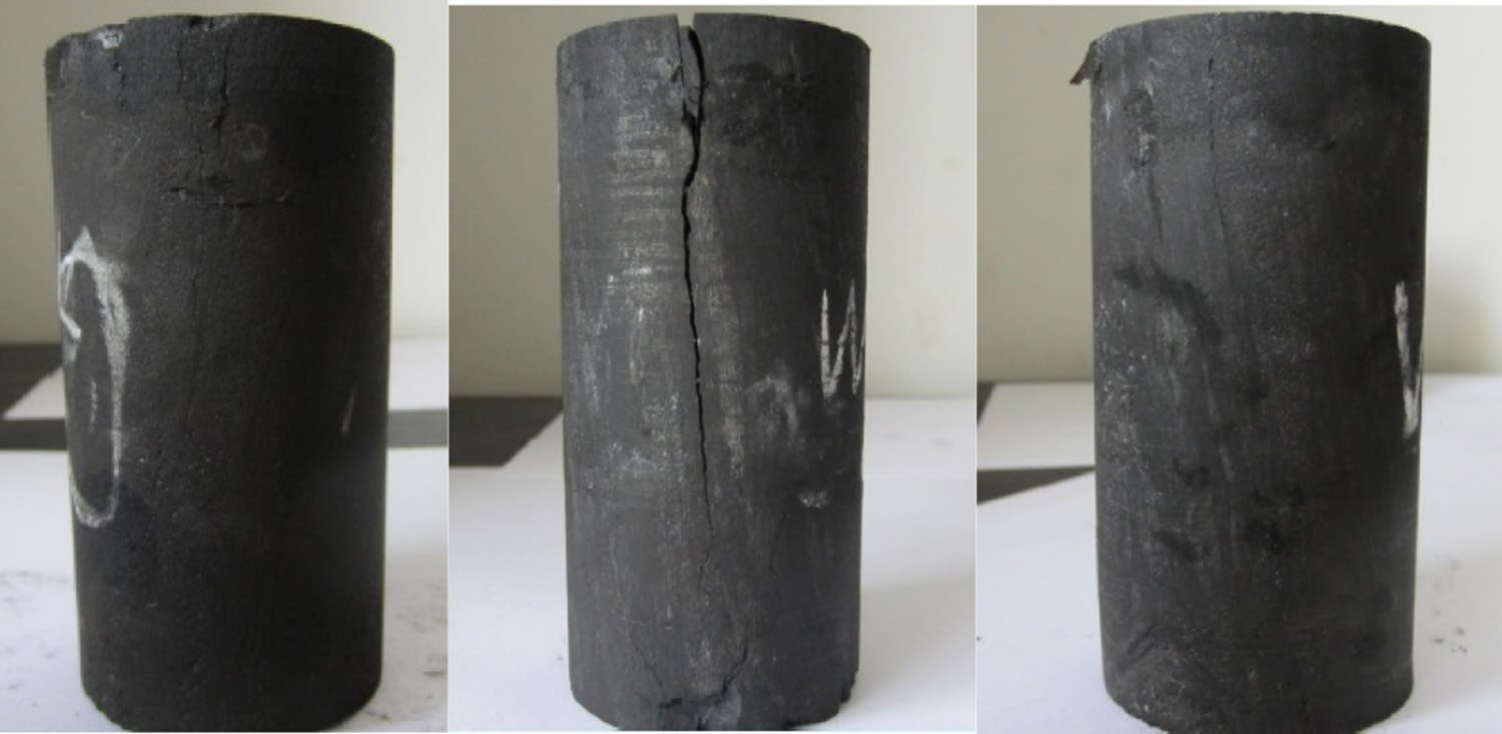
Failure modes of coal samples G1, W1 and W3.

The failure pressures of G1, W1 and W2 are 1.17, 1.2 and 0.41 MPa respectively. In practical engineering, the initial water injection pressure is primarily influenced by the internal fracture properties of the coal seam, as well as the pressure exerted by coalbed methane^24^. Extensive empirical evidence has demonstrated that the initial water injection pressure for coal seam water injection is typically quite low, typically below 3MPa. Experimental evidence demonstrates that the coal sample initially attains ultimate failure of tensile stress intensity due to the combined influence of water pressure and air pressure. Subsequently, under the force of pressure, the failure propagates along the crack surface, resulting in the formation of through cracks, as shown in Fig. 15.

## Numerical simulation of gas fracturing fluid–solid coupling

The phenomenon of fluid–solid coupling is characterized by a collection of interconnected equations that encompass both the fluid and solid domains. These equations involve unknown variables that encompass both fluid and solid phenomena. Specifically, two key characteristics are observed:

1. The solution of the fluid domain and the solid domain cannot be obtained independently; they are interdependent.
2. Neither the independent variables describing fluid motion nor those describing solid phenomena can be explicitly eliminated.

### Heterogeneity of rock mass materials

To capture the inherent heterogeneity of rock mass materials, a discretization technique that divides the chosen rock mass into finite elements for the purpose of numerical simulation is employed. The mechanical parameters of the rock mass, including Young's modulus and strength, are characterized by a Weibull distribution, which is described by the following probability density function:

30 $$ f(u) = \frac{m}{{u_{0} }}(\frac{u}{{u_{0} }})^{m - 1} \exp ( - (\frac{u}{{u_{0} }})^{m} ) $$

In this equation, the parameters of the unit cell are denoted by $u$, the size parameter $u_{0}$ is associated with the average value of the material parameters, and the shape parameter $m$ serves as an indicator of uniformity.

### Mechanical equilibrium and damage evolution equations of rock mass

During the simulation of pneumatic fracturing, it is postulated that the loading of the fractured rock mass occurs in two stages. The first stage involves the application of a short-duration dynamic stress wave, followed by the second stage characterized by a long-duration quasi-static gas pressurization. To account for this loading scenario, the modified Navier equations are employed to describe the mechanical equilibrium of the rock under dynamic loading and quasi-static pressure.

31 $$ G_{{u_{i,jj} }} + \frac{G}{1 - 2\upsilon }u_{j,ji} + \alpha p_{,i} + F_{i} = \rho_{s} \frac{{\partial^{2} u_{i} }}{{\partial t^{2} }} $$

The equation consists of various parameters. *G* denotes the shear modulus, representing the displacements occurring in different directions, $\upsilon$ signifies the Poisson's ratio of the material, $\alpha$ is the Biot coefficient, $F_{i}$ indicates the components of the body force in different directions, $\rho_{s}$ stands for the density of the solid, $t$ represents time. During the dynamic loading phase, the coefficient $\alpha p_{i}$ is assigned a value of zero. Similarly, during the quasi-static gas pressurization phase, the coefficients representing air pressure, denoted by $p$ and $\rho_{s} \frac{{\partial^{2} u_{i} }}{{\partial t^{2} }}$ are set to zero. Furthermore, in the process of gas expulsion following high-pressure air blasting and fracturing, the coefficient representing gas pressure $p$ as well as the right-hand term of the equation $\rho_{s} \frac{{\partial^{2} u_{i} }}{{\partial t^{2} }}$ are also rendered as zero. Under these specific circumstances, due to the significantly sluggish flow velocity of the coal seam gas, numerical analysis can solely be conducted within the quasi-static stage.

In this numerical simulation, the maximum tensile stress criterion and the Mohr–Coulomb strength criterion to evaluate the extent of tensile and shear damage in brittle media are employed, with a particular focus on shale gas reservoirs. The calculations can be performed based on the equation presented below:

32 $$ F_{1} = \sigma_{1} - f_{{_{t0} }} = 0 {\text{ or }}F_{2} = - \sigma_{3} + \sigma_{1} \frac{1 + \sin \varphi }{{1 - \sin \varphi }} - f_{c0} = 0 $$

In the event of damage and failure occurring in a rock mass, it is observed that the elastic modulus undergoes a concurrent variation, depicted by the equation:

33 $$ E = (1 - D)E_{0} $$

**Figure 16:**
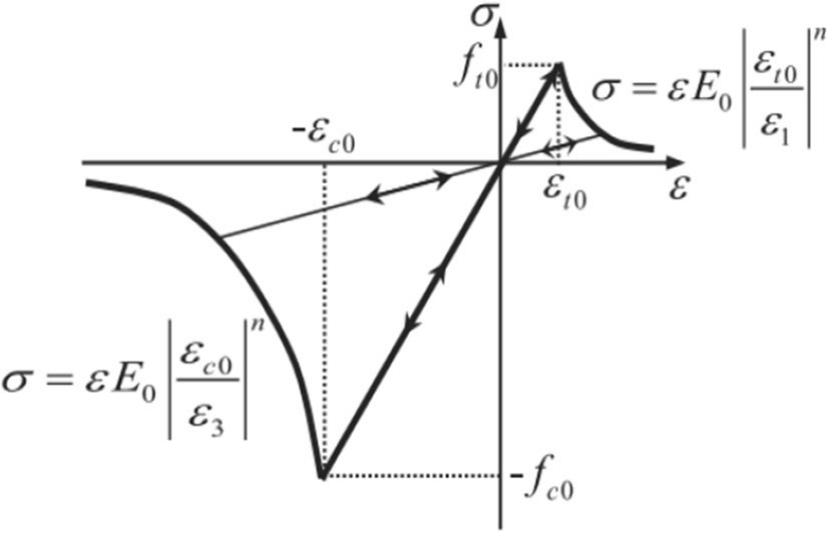
Element constitutive relation based on elastic damage in uni-axial stress conditions.

Moreover, based on the constitutive relationship diagram depicted in Fig. 16, the damage variable *D* can be defined using the following equation:

34 $$ D = \left\{ \begin{gathered} 0,F_{1} < 0\, \, and\, \, F_{2} < 0 \hfill \\ 1 - \left| {\varepsilon_{t0} /\varepsilon_{1} } \right|^{n} ,F_{1} = 0\, \, and\, dF_{1} > 0 \hfill \\ 1 - \left| {\varepsilon_{c0} /\varepsilon_{3} } \right|^{n} ,F_{2} = 0\, and\, dF_{2} > 0 \hfill \\ \end{gathered} \right. $$

### Gas equilibrium equation

Shale gas reservoirs are perceived as porous media comprising of pore spaces and solid matrix. In the course of gas fracturing, the infiltration of high-pressure air into the fractures and pores induces the expansion of pre-existing fractures.

The flow of gas within porous media is constrained by the gas mass equilibrium equation:

35 $$ \frac{{\partial (\phi \rho_{g} )}}{\partial t} + \nabla  \cdot (\rho_{g} q_{g} ) = Q_{s} $$

The density of the gas can be derived using the subsequent equation.

36 $$ \rho_{g = } \frac{p}{{p_{a} }}\rho_{ga} $$

Within this equation, $p_{a}$ and $\rho_{ga}$ designate the gas pressure and gas density under standard conditions, respectively.

Within our numerical simulations, it is assumed that the impact of gravity is overshadowed by the effects induced by pressure variations. Consequently, the Darcy velocity can be deduced from the following equation:

37 $$ q_{g} = - \frac{k}{\mu }\nabla p $$

In this equation, the symbol $k$ represents the permeability of the medium, while $\mu$ denotes the dynamic viscosity of the gas. By substituting Eqs. (36) and (37) into Eq. (35),

38 $$ \phi \frac{\partial p}{{\partial t}} - \nabla  \cdot \left(\frac{k}{\mu }p\nabla p\right) = \frac{{p_{a} }}{{\rho_{ga} }}Q_{s} $$

Equation (38) serves as a descriptor for the flow state of gas during the hydraulic fracturing process under high pressure.

For gas found within shale gas reservoirs, variations in gas pressure and porosity result in the occurrence of methane adsorption or desorption. When accounting for the adsorption or desorption of methane gas, the gas flow equation within the reservoir can be formulated as follows.

39 $$ \beta [\frac{\phi }{p} + \frac{{2a_{1} a_{2} \rho_{ms} }}{{1 + a_{2} p}} - \frac{{a_{1} a_{2}^{2} \rho_{s} p}}{{(1 + a_{2} p)^{2} }}]\frac{{\partial p^{2} }}{\partial t} - \nabla (\beta \frac{k}{\mu }\nabla p^{2} ) = Q_{S} $$

Within this equation, $\beta$ denotes the compressibility factor, while $a_{1}$ and $a_{2}$ signify the Langmuir volume constant and pressure constant.

The relationship between stress and porosity in a gas reservoir can be expressed as follows:

40 $$ \phi = (\phi_{0} - \phi_{r} )\exp (\alpha_{\phi }  \cdot \sigma_{\upsilon } ) + \phi_{r} $$

Within this equation, $\phi_{0}$ represents the initial porosity at zero stress, while $\phi_{r}$ indicates the porosity of the rock mass under high pressure. $\alpha_{\phi }$ represents the stress sensitivity coefficient of the rock mass, and $\sigma_{v}$ denotes the average effective stress.

Furthermore, the extent of damage to the rock mass also exerts an influence on its permeability, which can be evaluated through the application of the following formula:

41 $$ k = k_{0} (\phi /\phi_{0} )^{3} \exp (\alpha_{k} D) $$

where $k_{0}$ corresponds to the permeability at zero stress conditions, whereas $\alpha_{k}$ denotes the coefficient responsible for the permeability effect induced by damage.

To summarize, when subjecting the unit cell to dynamic loading, Eq. (31) characterizes the dynamic stress state during such loading, whereas Eq. (34) is employed to model the damage evolution process within the unit cell. Subsequently, the calculated damage variables, along with key parameters such as elastic modulus, material strength, and permeability, are utilized in the subsequent numerical analysis to simulate the quasi-static gas pressurization phase. In this phase, Eqs. (38) and (31) are employed to compute the quasi-static air pressure and stress state when the term $\rho_{s} \frac{{\partial^{2} u_{i} }}{{\partial t^{2} }}$ in Eq. (31) becomes zero. Furthermore, Eq. (34) is again employed to evaluate the damage evolution of the unit cell. Ultimately, the computed parameters such as effective porosity and permeability, as well as the distribution of damage obtained during the quasi-static phase, are transferred to the numerical analysis of gas extraction. Equations (38) and (39) are then utilized to model the gas extraction process.

### Model establishment

**Figure 17:**
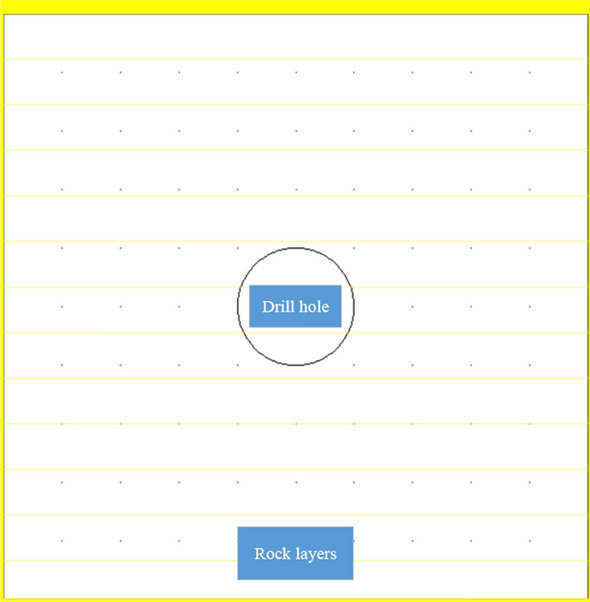
Establishment of a numerical simulation model for gas-pressure-induced fracturing in rock specimens.

The numerical simulation software is COMSOL Multiphysics, whose numerical principle is the finite element method. The advantage of this simulation software is that it uses finite element method to solve partial differential equations. Compared with finite difference method and spectral method to solve partial differential equations, more complex geometric structures and complex boundary conditions can be considered. In addition, COMSOL Multiphysics numerical simulation software has a complete interface with MATLAB and other software, which is convenient for post-processing of simulation data. Therefore, the COMSOL Multiphysics is utilized to construct a model that accurately represents the internal conditions of shale formations during gas-pressure-induced fracturing. A cube-shaped domain with a central borehole of radius *r* = 0.1 m is employed as the geometric representation of the reservoir, as shown in Fig. 17.

To simulate the pressure exerted on the model units by the surrounding rock mass, uniform pressure distributions are applied along the perimeter of the reservoir. Additionally, the borehole boundary is subjected to pressure to simulate the gas pressure experienced after high-energy gas explosions.

Considering the influence of temperature, certain regions of the model units representing the surrounding rock outside the borehole are assigned specific temperatures to represent the current temperature of the reservoir. Since the borehole remains connected to the external environment after fracturing, the temperature in the vicinity of the borehole is set to ambient temperature.

After finalizing the boundary conditions, various parameters are assigned to the simulated rock units in order to accurately represent the shale formation. These parameters include the solid density of the rock, density of the shale gas, porosity of the formation, permeability, as well as temperature profiles for both the rock and the external environment. Additionally, mechanical properties such as Young's modulus are considered to capture the mechanical behavior of the rock.

### The development of rock damage under pneumatic fracturing

To conduct a comprehensive analysis of the fluid–solid coupling mechanics in shale formations subjected to pneumatic fracturing, it is essential to simulate the development of fractures within the rock layers after pneumatic fracturing. Only after obtaining a clear understanding of the evolution of these fractures, the data processing and analysis can be proceed.

**Figure 18:**
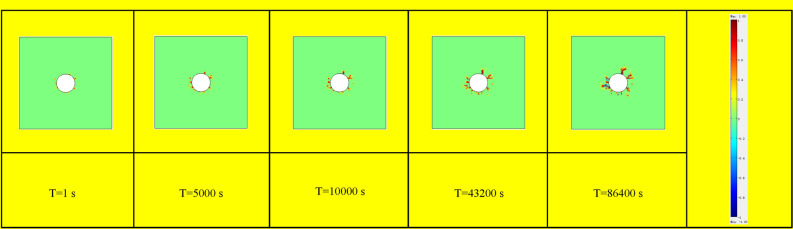
Simulated fracture damage development diagram of rock specimens.

To simulate the impact of pneumatic fracturing pressure, a designated unit cell is used to apply a specific pressure around the borehole. Given that the rock formation naturally contains fractures that enhance gas permeability, the introduction of high-pressure gas leads to pressurization in the vicinity of these existing cracks. This results in a gradual enlargement of fractures during the fracturing process, eventually leading to complete rock mass failure. The progression of fractures in the rock mass surrounding the borehole, simulated using specialized software, depicted in Fig. 18.

It is evident that the fractures within the rock mass gradually propagate along the periphery of the borehole, mimicking the internal expansion of the rock layers, under the influence of high-pressure gas. This propagation continues until the specimen is ultimately fractured under compression.

During the progressive development of damage in pneumatic fracturing of rock formations, several relevant physical and mechanical parameters within the rock layers undergo noticeable changes as the damage evolves. By analyzing the selected parameters at different time steps during the damage simulation process, it is observed that these parameters exhibit corresponding variations in response to the evolving damage within the rock layers.

**Figure 19:**
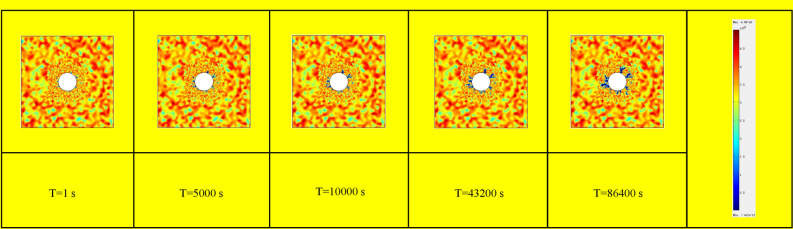
Elastic modulus change with time during damage process.

Figure 19 illustrates the fluctuations of the elastic modulus within the unit cell. In these figures, positive stress values correspond to tension, while negative values denote compression. After simulating the aforementioned cloud maps, several observations can be made. Firstly, pneumatic fracturing indeed exerts significant stress on the surrounding borehole rock formations, causing the strain around the borehole to gradually increase with the progression of the damage process until failure occurs. Secondly, during the process of damage evolution, the permeability of the rock mass gradually increases, facilitating the movement of shale gas and thereby facilitating its extraction.

## Conclusions

1. The analysis of unsteady seepage reveals that the pore pressure gradient at the coal entrance reaches its peak during the sudden increase in water pressure, gradually decreasing over time. Conversely, the pore pressure gradient at the exit side gradually increases. The most critical period of hydraulic fracturing is observed during the sudden surge in water pressure at the water inlet. Prolonged exposure to this elevated hydraulic gradient heightens the risk of hydraulic fracturing.
2. The duration needed for seepage to reach stability directly correlates with the length of time during which a high hydraulic gradient is sustained. Consequently, prolonged exposure to this elevated hydraulic gradient heightens the risk of hydraulic fracturing. The triaxial rock mechanics test system and coal pneumatic fracturing equipment are used to carry out the hydraulic and pneumatic fracturing experiment of raw coal. The failure mechanism of experimental coal samples is analyzed by employing the principles of elastic mechanics and fracture mechanics.
3. The evolution of fractures in coal samples can be classified into three distinct stages: the initial stage characterized by negligible volume changes despite increasing water pressure and stress, the expansion stage wherein pressures reach the failure strength and fractures propagate, and the fracture closure stage wherein unloading pressures cause minimal or even negative changes in volume. Acoustic emission signal can well correspond to these three stages. At the same time, the diametral tensile failure mode of coal sample also verifies the initial failure and crack propagation mode in the theoretical analysis.

## Data Availability

Some or all data, models, or codes generated or used during the study are available from the corresponding authors by request.
